# Supplementary material for: Triple-Helical DNA in Drosophila Heterochromatin
Source: Cells. 2018 Nov 23;7(12):227. doi: 10.3390/cells7120227 (PMC6316130; doi:10.3390/cells7120227)
Supplement: Supplementary file 1 [file cells-07-00227-s001.pdf]

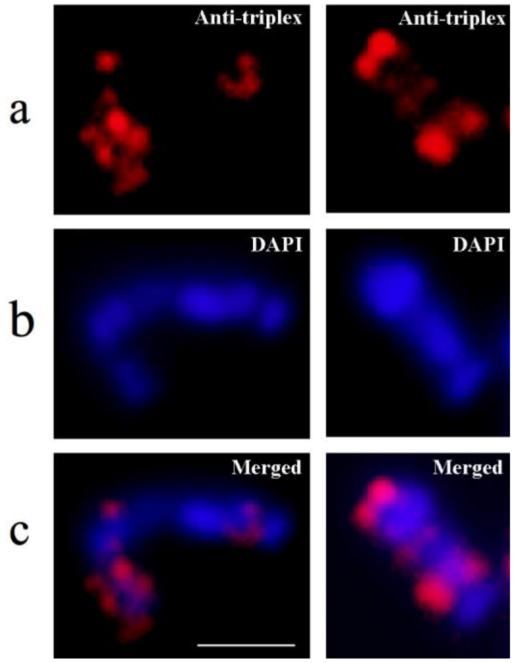

Figure S1. Specific regions in the Y chromosome of *D. melanogaster* are stained by anti-triplex antibodies. Antibody labelling in neuroblast chromosomes (**a**, **anti-triplex)**. DAPI staining (**b**, **DAPI**) and the corresponding merged signals (**c**, **Merged)**. Scale bar corresponds to 5 μm.

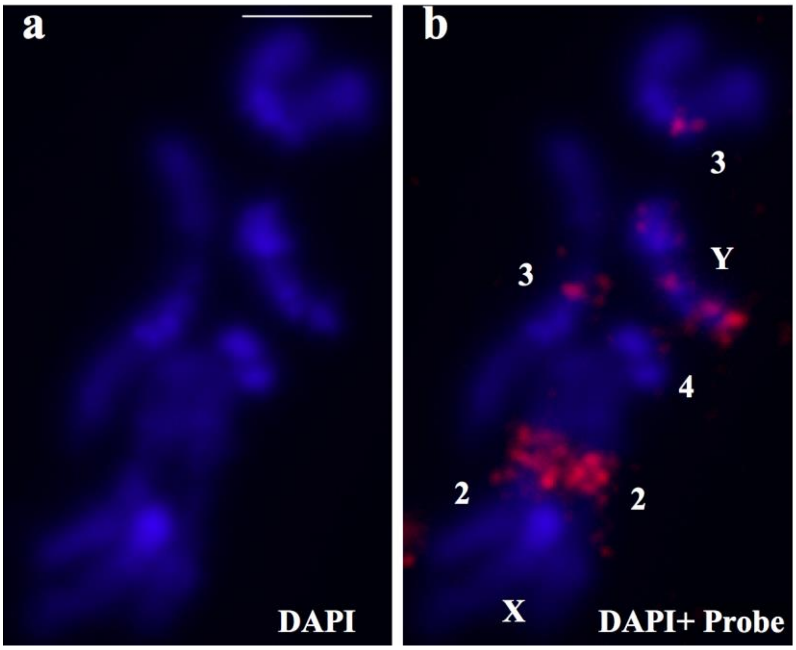

Figure S2. Localisation of AAGAG repeats in mitotic chromosomes of *D. melanogaster*. *In situ* hybridisation was performed with neuroblast chromosomes. Chromosomal DNA stained with DAPI (**a**, **DAPI**), satellite probe labelling (red signal) and DAPI staining superimposed (**b**, **DAPI+ probe**). Chromosomes appear identified (2, 3, 4, X, Y). Scale bar corresponds to 5 μm.

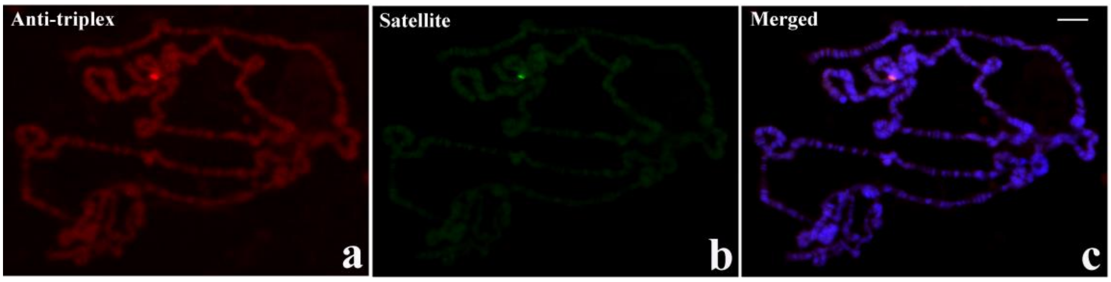

Figure S3. Anti-triplex DNA detection followed by localization of AAGAG repeats in polytene chromosomes of *D. melanogaster* carrying the bw^D allele. Anti-triplex DNA labeling (**a**, **Anti-triplex**), satellite probe labelling (**b**, **Satellite**) and the corresponding merged signals together with DAPI staining, (**c**, **Merged**). Scale bar corresponds to 20 μm.

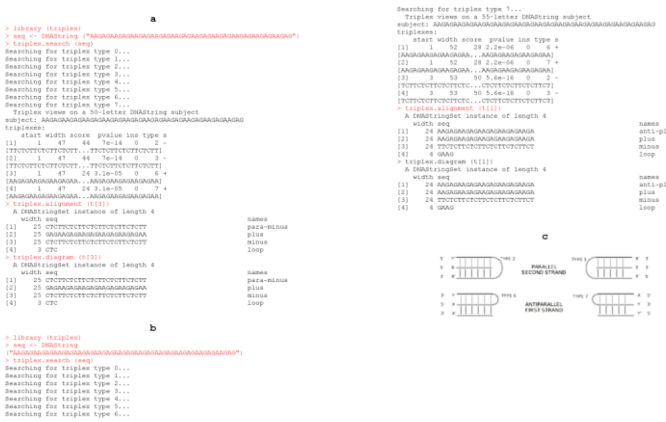

Figure S4. Some triplex DNA outputs are shown, as part of functions described in "Triplex" software package user guide. Triplex types (2, 3, 6, 7) are identified after loading "library (triplex)", AAGAG tandem repeats and performing "triplex-search" function. Triplex DNA visualisation is achieved by "triplex-alignment" and "triplex-diagram" functions (**a**, **b).** "Triplex-diagram" display figures as shown in Figure 3. Triplex type figures (2, 3, 6, 7) obtained after loading AAGAG tandem repeats as depicted in the user guide (**c**).

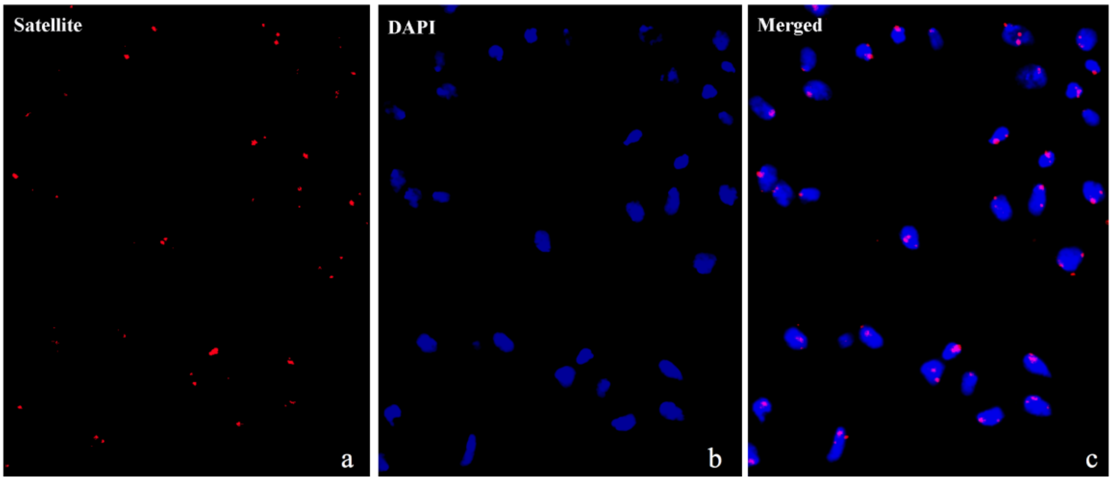

Figure S5. Satellite probe hybridisation to *Drosophila* neuroblast nuclei prepared in pH 7.0 omitting chromosomal DNA denaturation. Neuroblast nuclei carrying the bw^D allele are

shown. AAGAG probe labeling (**a**, **Satellite**), chromosomal DNA staining (**b**, **DAPI**) and the corresponding merged signals (**c**, **Merged)**.

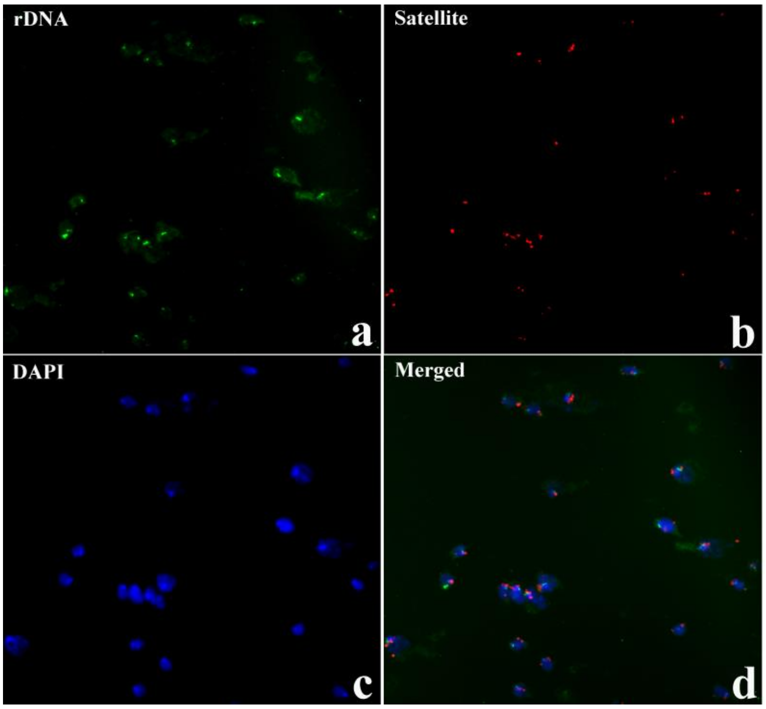

Figure S6. Ribosomal DNA and AAGAG probe hybridisation to *Drosophila* neuroblast nuclei carrying the bw^D allele prepared in pH 7.0 after denaturing chromosomal DNA. Ribosomal DNA probe labelling (**a**, **rDNA**), AAGAG probe labelling (**b**, **Satellite**), nuclei stained with DAPI (**c**, **DAPI**) and the corresponding merged signals (**d**, **Merged**).
